# *F. prausnitzii* and its supernatant increase SCFAs-producing bacteria to restore gut dysbiosis in TNBS-induced colitis

**DOI:** 10.1186/s13568-021-01197-6

**Published:** 2021-02-28

**Authors:** Youlian Zhou, Haoming Xu, Jing Xu, Xue Guo, Hailan Zhao, Ye Chen, Yongjian Zhou, Yuqiang Nie

**Affiliations:** 1 Department of Gastroenterology, Guangzhou Digestive Disease Center, Guangzhou First People’s Hospital, School of Medicine, South China University of Technology, No. 1 Panfu Road, Guangzhou, 510180 China; 2 Department of Gastroenterology, Nanfang Hospital, Southern Medical University, Guangzhou, 510515 China

**Keywords:** Inflammatory bowel disease, Gut microbiota, *F. prausnitzii*, Supernatant, Short chain fatty acid

## Abstract

An increasing number of studies have shown that *Faecalibacterium prausnitzii* (*F. prausnitzii*) is a promising anti-inflammatory bacterium that colonizes in the gut and that gut microbiota dysbiosis plays an important role in the pathogenesis of inflammatory bowel disease (IBD). In this study, we report the gut microbiota profile of 2,4,6-trinitrobenzenesulfonic acid (TNBS)-induced colitis mice treated with *F. prausnitzii* and its supernatant on the basis of high-throughput sequencing. We interestingly found that both *F. prausnitzii* and its metabolites exerted protective effects against colitis in mice, which ameliorated gut dysbiosis, with an increase in bacterial diversity and the abundance of short-chain fatty acid (SCFA)-producing bacteria and a decrease in serum TNF-α and the abundance of Proteinbacteria, Acidobacteria, and Bacteroidetes. These findings will provide further evidence of the anti-inflammatory effect of *F. prausnitzii*, which presents therapeutic potential for IBD treatment.

**Supplementary Information:**

The online version contains supplementary material available at 10.1186/s13568-021-01197-6.

## Introduction

Inflammatory bowel disease (IBD) is a group of refractory digestive diseases including Crohn's disease (CD) and ulcerative colitis (UC). At present, there is a lack of satisfactory targeted drugs and therapies for the IBD. A large proportion of patients show complications or cancer development or require surgical treatment after using 5-aminosalicylates (5-ASA), steroids, immunomodulators, biological agents and other drugs. Compared with other gastrointestinal diseases, widespread concern about IBD has been aroused because of the high cost of its treatment and high relapse rate. To date, however, the pathogenesis of IBD has not been fully elucidated. It is well known that the gut microbiota plays a substantial role in the pathogenesis of IBD. There is increasing interest in the development of microbiota-modulating therapies for IBD, such as the use of fecal microbiota transplantation (FMT), probiotics and prebiotics.

*Faecalibacterium prausnitzii* (*F. prausnitzii*), is an anaerobic bacterium that mainly exists in the ileocecal and terminal ileum. Accumulating studies have shown that the abundance of *F. prausnitzii* is significantly reduced in the stool of patients with acute IBD, especially in those with acute UC, but there is no difference in the proportion of *F. prausnitzii* between patients in IBD remission and healthy controls (Sokol et al. 2009). Decreases in the abundance of *F. prausnitzii* in the intestine are significantly related to the activity of CD (Friswell et al. 2010; Sokol et al. 2008). A reduced abundance of *F. prausnitzii* in the intestinal mucosa of CD patients is positively correlated with the degree of intestinal lesions during surgery (Sokol et al. 2008). Sokol et al*.* (2009) compared the fecal microbiota of patients with acute CD, those in CD remission and patients with UC and found that the abundance of Firmicutes [*Clostridium leptum* and *Clostridium coccoides*] and the ratio of the *Firmicutes/Bacteroides* phyla were reduced in the stool of patients with acute IBD and infectious enteritis. After recurrence in patients with UC, if the abundance of *F. prausnitzii* in the intestine is restored, it will be beneficial to disease remission (Varela et al. 2013). In animal models and in vitro, *F. prausnitzii* may regulate the metabolites of the gastrointestinal tract and peripheral blood to reduce the occurrence of colitis (Miquel et al. 2015). In addition to its direct anti-inflammatory effects (Friswell et al. 2010), *F. prausnitzii* improves the permeability of the intestinal epithelium in an IBD rat model induced by dextran sodium sulfate (DSS) (Carlsson et al. 2013), which further confirms the positive protective effect of *F. prausnitzii* on IBD. While the investigation of *F. prausnitzii* has gradually become a research focus and hotspot, its mechanism in IBD is still unclear. Therefore, it is of great scientific significance and clinical value to explore the molecular mechanism of *F. prausnitzii* in IBD. In this study, we aimed to investigate the effect of *F. prausnitzii* on the gut microbiota and to evaluate its protective ability when therapeutically used in acute TNBS-induced murine colitis.

## Materials and methods

### Bacteria culture

*Faecalibacterium prausnitzii* (*F. prausnitzii*, ATCC27766), kindly provided by Professor Chenggong Yu from Nanjing Medical University, was grown in LYHBHI medium [(brain heart infusion (37 g/L), yeast extract (5 g/L), blood crystal (5 mg/L)], plus maltose (1 g/L), cellobiose (1 g/L), cysteine (0.5 g/L) at 37 °C in an anaerobic incubator (97% carbon dioxide and 3% hydrogen). According to the optical density (OD) at 600 nm, the growth curve and live bacteria count were calculated based on the absorbance value in combination with the bacterial plate count (Qiu et al. 2013; Sokol et al. 2008). The *F. prausnitzii* supernatant was collected from bacterial cultures at 1 × 10^9^ CFU/mL.

### Animal model

Sixty 8- to 10-week-old male mice were purchased from Guangdong Medical Laboratory Animal Center (GMLAC, Foshan, Guangdong, China). These mice were kept under SPF conditions according to relevant laws and regulations of the laboratory animal ethics committee of GMLAC.

The mice were divided into 5 groups (N = 12 per group): EtOH control (EtOH), TNBS (TNBS), TNBS + 5-ASA (TNBS + ASA), TNBS + *F. prausnitzii* (TNBS + Fp), and TNBS + *F. prausnitzii* supernatant (TNBS + SN). Four groups were induced to develop colitis via the intrarectal administration of TNBS (Sigma Aldrich), which was dissolved in 50% ethanol at a dose of 25 mg/L for administration at 0.1 mL/per mouse. Before 24 h of colitis induction, 5-ASA (Sigma, 7.5 mg/mL, 0.1 mL/10 g body weight), *F. prausnitzii* (2–4 × 10^10^ CFU/kg), the *F. prausnitzii* supernatant (0.1 mL/10 g body weight) and PBS (0.1 mL/10 g body weight) were administered intragastrically to the mice once, which was repeated daily for the next 7 days. The EtOH control mice received PBS orally once in the first 24 h and on each of the following 7 days and were injected with 0.1 mL of a 50% ethanol solution in the rectum once in the second day. The experiment ended 24 h after the last intervention.

### Colonic pathology

At the end of the experiment, 6 mice were randomly selected from each group. Their fresh colon tissues were collected, washed, fixed in a 4% neutral formaldehyde solution for 24 h, and routinely dehydrated, embedded, cut into 4 μm sections, which were then stained with hematoxylin–eosin (H–E). The colonic histopathological score was assessed blindly using a previously published grading system (Fuss et al. 2002).

### TNF-α detection in mouse serum

TNF-α concentrations in mouse serum were quantified with an ELISA kit (Abcam, ab100747) according to the manufacturer’s recommendations.

### Stool sampling, short-chain fatty acids (SCFAs) detection and microbiota analysis

Stool samples from mice were collected by stimulating the anus and immediately frozen in a − 80 °C refrigerator. Fecal SCFAs were detected by gas chromatography–mass spectrometry (GC–MS) as previously described (He et al. 2020; Wang et al. 2019). Stool DNA extraction was performed with the Qiagen DNA Stool MiniKit (Qiagen, Cat No. 51504). PCR and 16S rRNA sequencing were conducted using a MiSeq System (Illumina, Inc.). Bioinformatic analysis was performed as previously described (Zhou et al. 2018b, c). For the statistical analysis, we used the unpaired Student’s t-test, Wilcox test and Mann–Whitney test to compare two groups. One-way ANOVA, the Kruskal–Wallis test and Tukey’s posttest were used for comparisons of three groups. The results were considered statistically significant when P < 0.05 (*P < 0.05, **P < 0.01, ***P < 0.005). All analyses were performed with Prism GraphPad 6.0 and SPSS 19.0 software.

## Results

### *F. prausnitzii* and its supernatant relieve symptoms and reduce body weight and colonic inflammation in mice

**Fig. 1:**
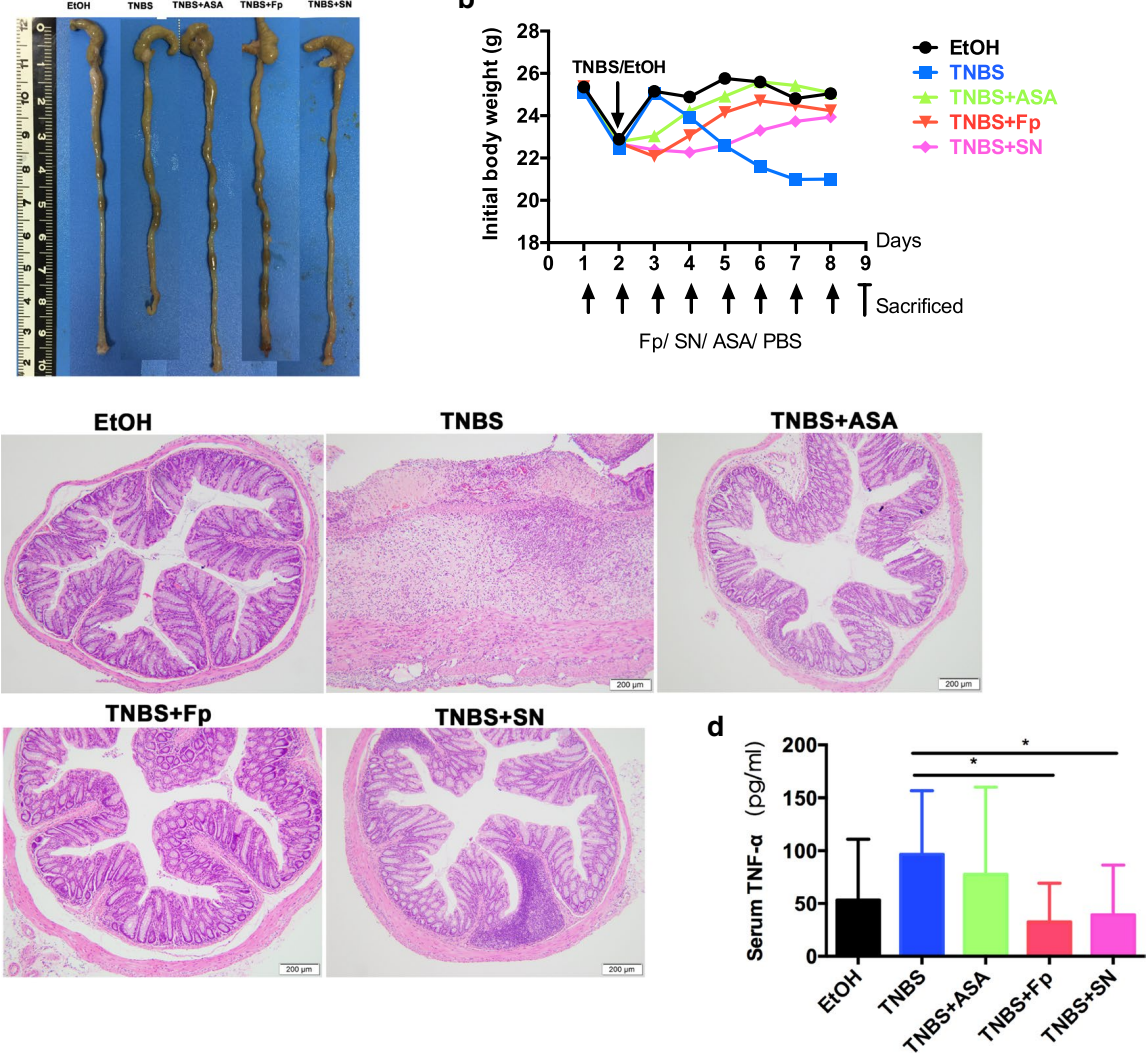
Effects of *F. prausnitzii* and its supernatant on intestinal inflammation in an enteritis mouse model. **a** Colon length; **b** body weight; **c** histological HE; **d** serum TNF-α concentration. Data are the mean ± SD. *P < 0.05

Mice became symptomatic, showing symptoms such as weight loss, loose stool, and bloody diarrhea by day 2–3 of intrarectal TNBS administration. The symptoms were significantly improved by *F. prausnitzii* and its supernatant. Compared with the control group, the mice in the TNBS model group exhibited a markedly shorter colon length. The gross observation of the bowel revealed that the intestines of the mice in the EtOH group were pink, smooth and intact, while the gut lumen of the mice from the TNBS group showed swelling, congestion, a thinner colon wall, disappearance of mucosal folds, and a shortened colon. In some segments, large amounts of loose, unshaped or bloody feces were deposited in the gut lumen. After longitudinal incision, obvious ulcers were observed, and a few of the specimens exhibited intestinal adhesions and intestinal obstruction. Most of the changes observed in the gross observation of the gut were mitigated by *F. prausnitzii* and its supernatant to some extent. Histological examination also showed that *F. prausnitzii* and its supernatant could also ameliorate mucosal inflammation and histopathologic damage compared with the TNBS model group. Compared with the TNBS model group, the serum TNF-α concentrations in *F. prausnitzii* (32.51 ± 16.41 vs. 96.55 ± 16.06) and its supernatant (39.27 ± 16.70 vs. 96.55 ± 16.06) were also significantly decreased (Fig. 1).

### *F. prausnitzii* and its supernatant increase gut microbiota diversity

**Fig. 2:**
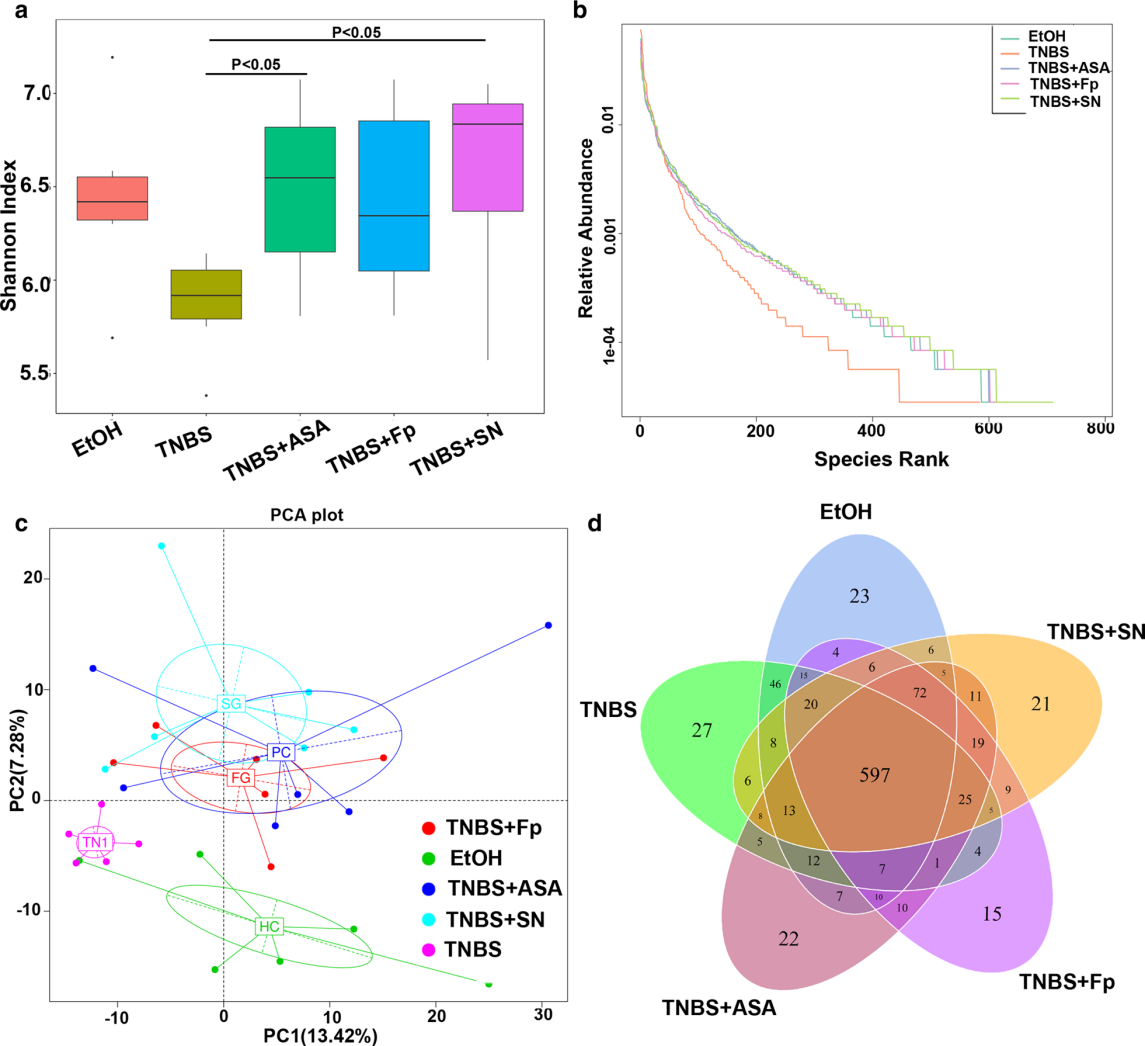
*F. prausnitzii* and its supernatant significantly improved the diversity of the gut microbiota in colitis mice. **a** Shannon index; **b** species rank abundance curve; **c** PCA; **d** Venn diagram

Mounting evidence shows that gut microbiota dysbiosis plays a significant role in the occurrence and development of IBD. To further study the effects of *F. prausnitzii* and its supernatant on the gut microbiota in IBD, we used 16S sequencing to further clarify the differences in the gut microbiome. Our findings indicated that the microbial diversity in the TNBS model group was significantly different from that in the EtOH control group, which was generally accompanied by a decrease in the diversity (Shannon index and species rank abundance curve) of gut microbiota. Treatment with both *F. prausnitzii* and its supernatant increased the diversity of the gut microbiota, especially in the supernatant group (P < 0.05). Based on the analysis of the number of OTUs, the 5 groups shared 597 OTUs, 23 of which were unique to the EtOH control group, while 27 were unique to the TNBS model group, and 22 were unique to the TNBS + ASA group. There were 15 and 21 unique OTUs in *F. prausnitzii* and its supernatant, respectively (Fig. 2).

### *F. prausnitzii* and its supernatant restore gut microbiota dysbiosis and increase the abundance of SCFA- producing bacteria

**Fig. 3:**
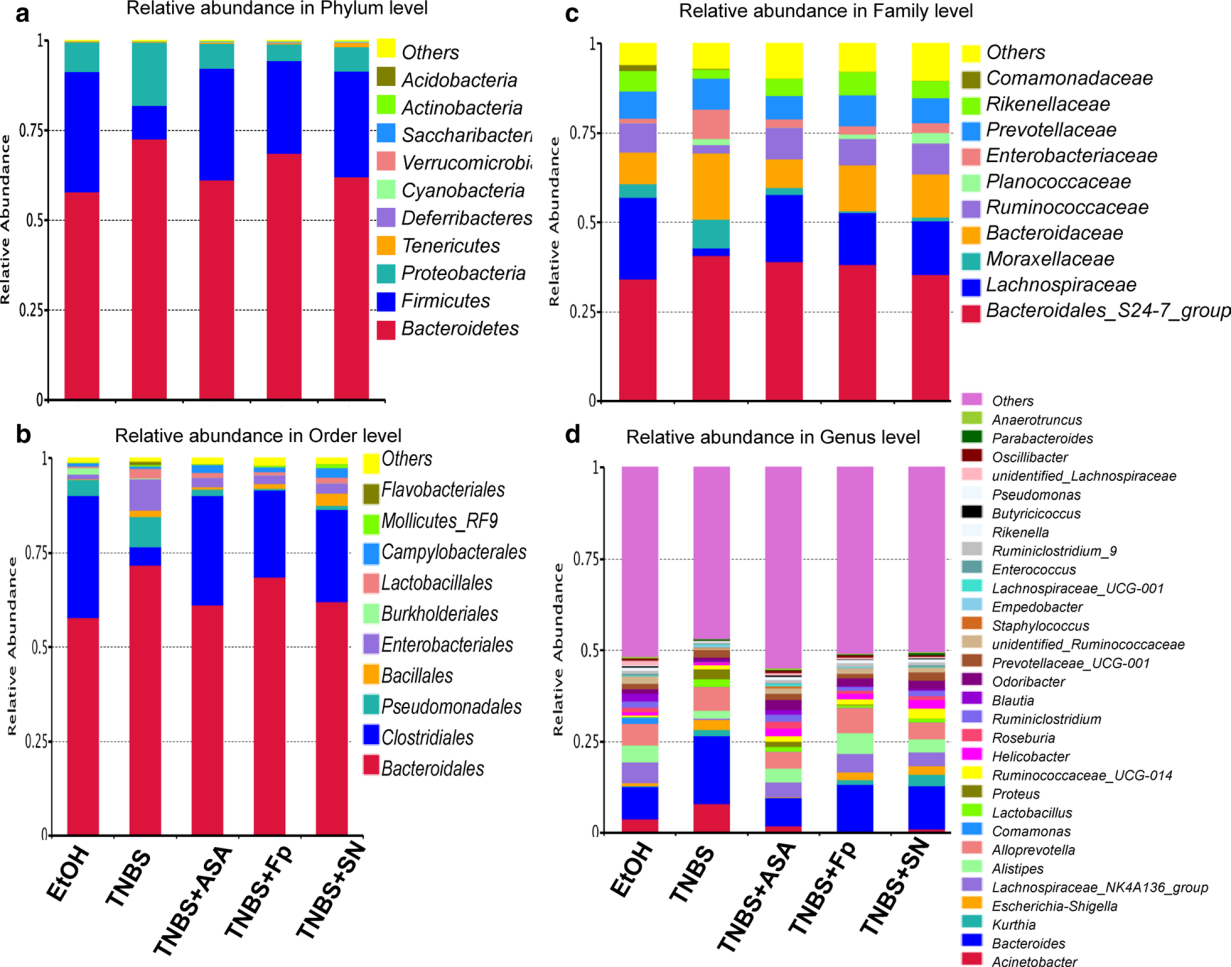
Histogram of taxon levels. **a** Phylum level; **b** order level; **c** family level, and **d** genus level

**Fig. 4:**
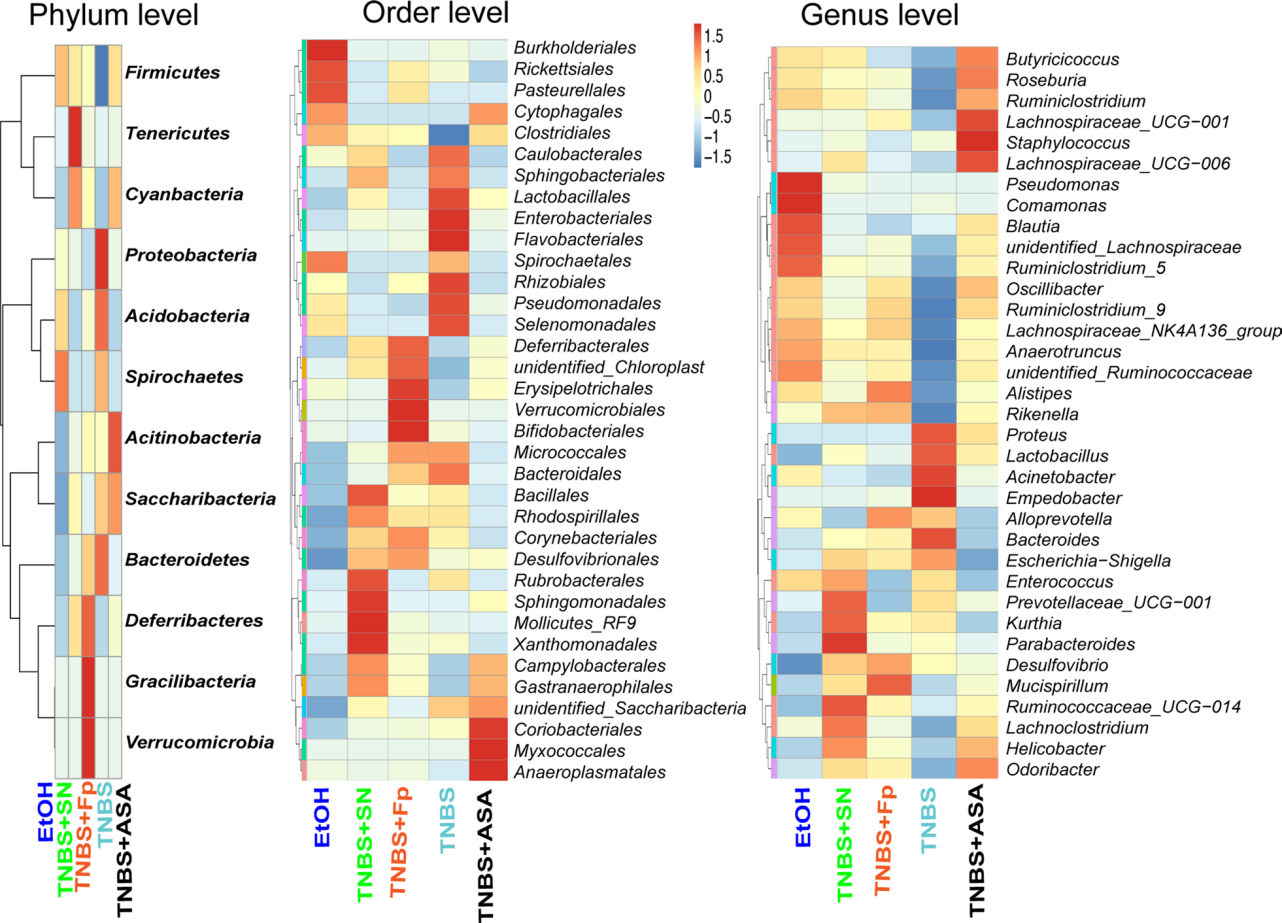
Microbial heatmap of the top OTUs at the phylum, order, and genus levels

**Fig. 5:**
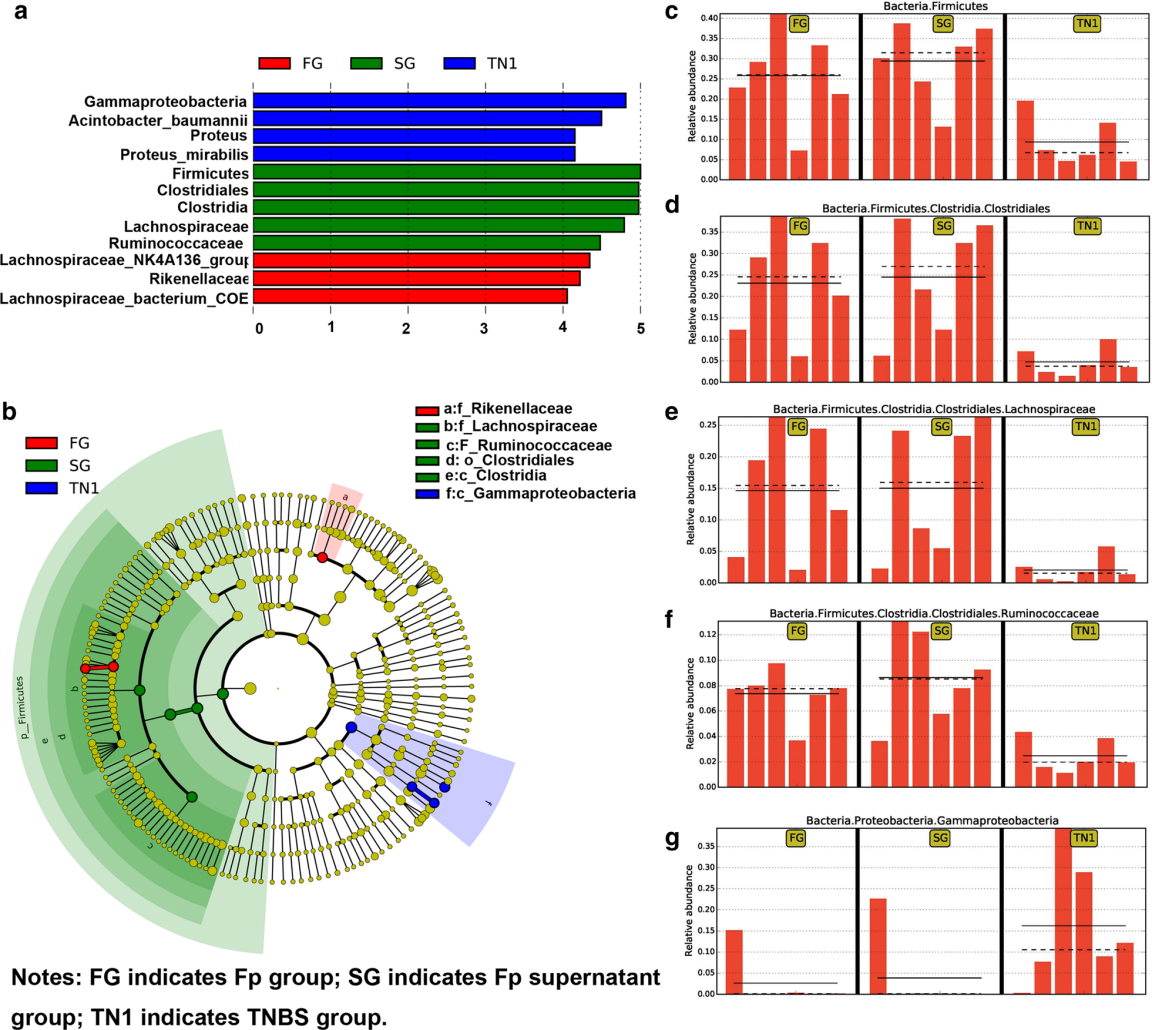
Microbial biomarkers in the TNBS model, *F. prausnitzii* and its supernatant groups. **a** Linear discriminant analysis (LDA) score; **b** LDA effect size (LEfSe), and relative abundance of **c** *Firmicutes*, **d** *Clostridiales*, **e** *Lachnospiraceae*, **f** *Ruminococcaceae*, and **g** *Gammaproteobacteria*

We further explored the changes in the gut microbiota composition, as shown by the relative abundance (Fig. 3), OTU level (Fig. 4) and LefSc biomarkers (Fig. 5). Compared with the TNBS model group, treatment with both *F. prausnitzii* and its supernatant increased the abundance of *Firmicutes*, *Cyanobacteria* and *Clostridiales*, and decreased the abundance of *Proteinbacteria*, *Acidobacteria*, and *Bacteroidetes*. At the genus level, *F. prausnitzii* increased the abundance of *Butyricicoccus*, *Roseburia*, *Ruminiclostridium*, *Lachnospiraceae*, *Oscillibacter*, *Anaerotruncus*, *Ruminococcaceae*, *Alistipes*, *Rikenella*, *Allspirophore*, *Deisulfoprecotella*, and *Mucispirllum*, which was more obvious in the *F. prausnitzii* supernatant group than in the TNBS model group. Notably, the *Butyricicoccus*, *Roseburia*, *Lachnospiraceae*, *Ruminococcaceae*, *Rikenella*, and *Eubacterium_xylanophilum* are short-chain fatty acid (SCFA)-producing bacteria.

**Fig. 6:**
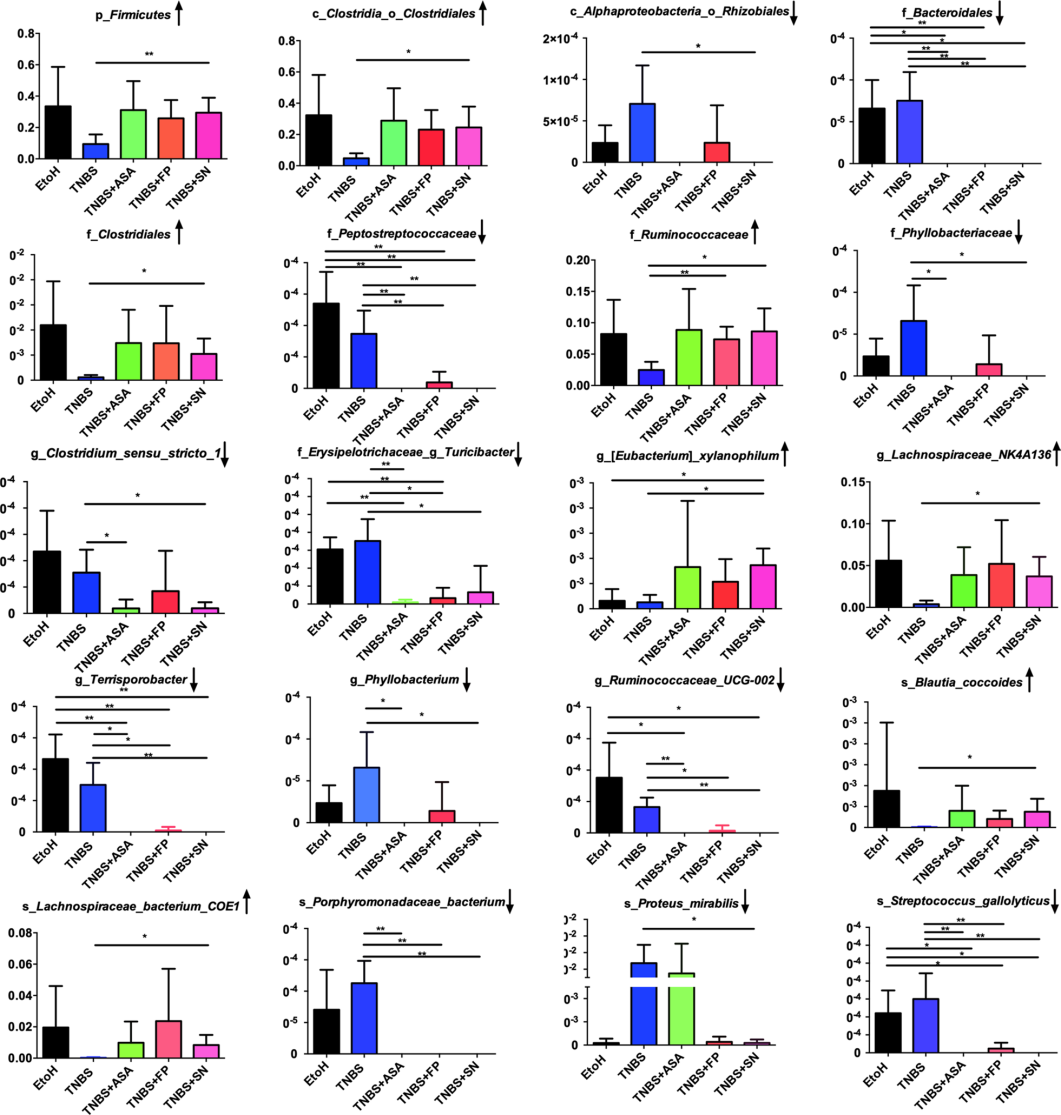
MetaStat analysis at different taxon classification levels. The X axis represents the sample groups, and the Y axis represents species abundance

Next, we investigated significant taxonomic shifts in the microbial community through MetaStats analysis. Compared with the TNBS model group, *F. prausnitzii* treatment significantly increased the abundance of *Ruminococcaceae* (P < 0.01) and decreased the abundance of *Peptostreptococcaceae* (P < 0.01),* Ruminococcaceae_UCG* (P < 0.05), *Terrisporobacter* (P < 0.05), and *Turicibacter* (P < 0.05). These changes were the most obvious in the *F. prausnitzii* supernatant group (TNBS + SN) and were characterized by increased the abundance of Firmicutes (P < 0.01), *Clostridiales* (P < 0.05), *Ruminococcaceae* (P < 0.05), *Clostridiales_vadinBB60* (P < 0.05), *Lachnospiraceae_NK4A136* (P < 0.05), *Eubacterium_xylanophilum* (P < 0.05), *Lachnospiraceae_bacterium_COE1* (P < 0.05), *Blautia_coccoides* (P < 0.05), and reducing the abundance of *Rhizobiales* (P < 0.05), *Peptostreptococcaceae* (P < 0.01), *Phyllobacteriaceae* (P < 0.05), *Ruminococcaceae_UCG-002* (P < 0.01), *Terrisporobacter* (P < 0.01), *Turicibacter* (P < 0.05), *Clostridium_sensu_stricto* (P < 0.05), *Phyllobacterium* (P < 0.05), *Proteus_mirabilis* (P < 0.05), *Streptococcus_gallolyticus* (P < 0.01), and *Porphyromonadaceae_bacterium* (P < 0.01) (Fig. 6). Interestingly, *Lachnospiraceae*, *Ruminococcaceae*, *Blautia* and *Eubacterium_xylanophilum* are also SCFAs-producing bacteria. In addition, to determine the alterations of SCFAs after treatment of *F. prausnitzii* and its supernatant, fecal SCFAs including acetic acid, propionic acid, butyric acid and amyl acid were detected. As shown in Additional file 1: Figure S1, both *F. prausnitzii* and its supernatant could increase the content of SCFAs in the feces compared with the TNBS model group, further indicating that *F. prausnitzii* and its supernatant increase the abundance of SCFA-producing bacteria.

## Discussion

Inflammatory bowel diseases (IBD) are multifactorial disorders characterized by a partially unclear etiology and pathogenic mechanisms including gut microbiota dysbiosis. Our previous study (Zhou et al. 2018c) showed that the fecal microbiota of humans provides promising universal and promising biomarkers for the evaluation of IBD severity and IFX treatment efficacy. The composition of the intestinal microbiome and microbial-derived metabolites seem to influence the healthy state of the host both by both regulating metabolism-related pathways and affecting the expression of different genes via epigenetic effects (De Musis et al. 2020). Based on this, it has been suggested that the gut microbiota might be a promising therapeutic target in IBD. Accumulating studies have suggested that probiotic administration is useful for IBD.

*Faecalibacterium prausnitzii*, a representative commensal bacterium of the Firmicutes phylum, can decompose undigested cellulose in the intestinal lumen to produce SCFAs such as butyric acid, which not only provide energy for intestinal mucosal epithelial cells, but also benefit the intestinal environment and the stability of the intestinal mucosal barrier. Sokol et al*.* (2009) confirmed that the administration of *F. prausnitzii A2-165* or its supernatant significantly decreased colitis severity induced by TNBS and restored gut microbiota dysbiosis, as demonstrated by real-time quantitative PCR (qPCR) analysis. *F. prausnitzii A2-165* also exerted anti-inflammatory effects on both cellular and colitis animal models by blocking NF-KB activation and IL-8 production (Sokol et al. 2009), facilitating the induction of IL-10 in human and murine dendritic cells and modulating T cell responses (Rossi et al. 2016). Furthermore, *F. prausnitzii* (strain *A2-165*) or its supernatant has been confirmed to induce a significant reduction in colitis severity by downregulating MPO, pro-inflammatory cytokines, and T-cell levels (Martin et al. 2014), and protecting the intestinal epithelial barrier(Carlsson et al. 2013; Martin et al. 2015) in colitis mouse models. *F. prausnitzii* (*ATCC 27766*) or its supernatant can also exert protective effects by inhibiting Th17 differentiation and IL-17A secretion in both DSS-induced mouse colitis (Huang et al. 2016) and TNBS-induced colorectal colitis in rats (Zhang et al. 2014, 2019), and upregulating regulatory T cells (Qiu et al. 2013) and butyrate production to maintain Th17/Treg balance (Zhou et al. 2018a) in a colitis rat model and in vitro. In addition, the *F. prausnitzii* HTF-F strain and the extracellular polymeric matrix (EPM) exerted anti-inflammatory effects on the clinical parameters measured in the DSS model (Rossi et al. 2015).

In this study, we report the gut microbiota profile of TNBS-induced colitis mice treated with *F. prausnitzii* (ATCC 27766) and its supernatant on the basis of high-throughput sequencing, and we found that treatment with *F. prausnitzii* and its supernatant significantly improved the colitis-related symptoms, increased the gut microbial diversity and mitigated gut dysbiosis*.* Our previous study (Zhou et al. 2018c) confirmed a relative decrease in SCFA-producing bacteria, including *F. prausnitzii*, *Lachnospiraceae*, *Ruminococcaceae* and *Roseburia*, which all belong to *Clostridiales* within the *Firmicutes* phylum, in patients with IBD. In this study, we interestingly found that the protective effects of *F. prausnitzii* and its supernatant may result from an increase in the abundance of SCFA-producing bacteria such as *Butyricicoccus*, *Roseburia*, *Lachnospiraceae*, *Ruminococcaceae*, *Rikenella*, *Eubacterium_xylanophilum* and *Blautia*, indicating the restoration of gut microbiota dysbiosis after *F. prausnitzii* or supernatant treatment. The mechanism behind the potential protective role of *F. prausnitzii* on gut microbiota may associate with competition for nutrients, metabolites and occupying effects to regulate commensal organisms and restrain pathogenic bacteria. It was confirmed that butyrate-producing bacteria belong to the *Firmicutes* phylum, mainly including *Clostridium leptum* and *Clostridium coccoides* (Kumari et al. 2013), which are mainly represented by *Faecalibacterium prausnitzii* and *Roseburia*, respectively (Louis and Flint 2009).

SCFAs are organic fatty acids with aliphatic tails of five or fewer carbons that are mainly produced by anaerobic microorganisms fermenting indigestible carbohydrates. In vitro studies of human intestinal cells have shown that butyrate can promote intestinal mucosal repair and inhibit the formation of inflammatory cytokines, thereby exerting an anti-inflammatory effect (Augenlicht et al. 2002; Segain et al. 2000). Through intestinal perfusion, sodium butyrate can also promote colonic mucosal healing in TNBS-induced colitis. In vitro studies have shown that butyrate also exhibits antitumor effects, including the reduction of the secretion of tumor necrosis factor (TNF) in intestinal epithelial cells (Segain et al. 2000), and induction of the differentiation and apoptosis of tumor cells, thereby inhibiting tumor growth (Pryde et al. 2002). In addition, SCFAs especially butyrate could regulate gut microbiota by modulating intestinal lumen pH values that are beneficial for SCFAs-producing bacteria (Canani et al. 2011; Guilloteau et al. 2010). Moreover, butyrate could preserve epithelial hypoxia and limit the overgrowth of nitrate respiration-dependent bacteria to maintain intestinal homeostasis (Byndloss et al. 2017; Winter et al. 2013).

In summary, this work showed that both *F. prausnitzii* and its metabolites exerted protective effects against colitis in mice, which ameliorated gut dysbiosis, with an increase in bacterial diversity and the abundance of SCFA-producing bacteria and a decrease in serum TNF- α and the abundance of *Proteinbacteria*, *Acidobacteria*, and *Bacteroidetes*. These findings will provide further evidence of the anti-inflammatory effect of *F. prausnitzii*, which presents therapeutic potential for IBD treatment.

## Supplementary Information

**Additional file 1: Figure S1.** The fecal SCFAs concentrations including acetic acid (A), propionic acid (B), butyric acid (C) and amyl acid (D).

## Data Availability

The data used to support the findings of this study are available in NCBI-SRA under accession number PRJNA669888 (https://www.ncbi.nlm.nih.gov/sra/?term=PRJNA669888).

## Abbreviations

IBD: Inflammatory bowel disease
TNBS: 2,4,6-Trinitrobenzenesulfonic acid
SCFAs: Short-chain fatty acids
CD: Crohn’s disease
UC: Ulcerative colitis
5-ASA: Five-aminosalicylates
FMT: Fecal microbiota transplantation
DSS: Dextran sodium sulfate
OD: Optical density
CFU: Colony forming unit
SN: Supernatant
SPF: Specific pathogen free
GMLAC: Guangdong Medical Lab Animal Center
PBS: Phosphate-buffered saline
ELISA: Enzyme linked immunosorbent assay
HE: Hematoxylin–eosin
ANOVA: Analysis of variance
OTU: Operational taxonomic unit
LDA: Linear discriminant analysis
LEfse: Linear discriminant analysis (LDA) effect size
EPM: Extracellular polymeric matrix
TNF: Tumor necrosis factor
