# Supplementary figures and images for: F. prausnitzii and its supernatant increase SCFAs-producing bacteria to restore gut dysbiosis in TNBS-induced colitis
Source: AMB Express. 2021 Feb 28;11:33. doi: 10.1186/s13568-021-01197-6 (PMC7914335; doi:10.1186/s13568-021-01197-6)

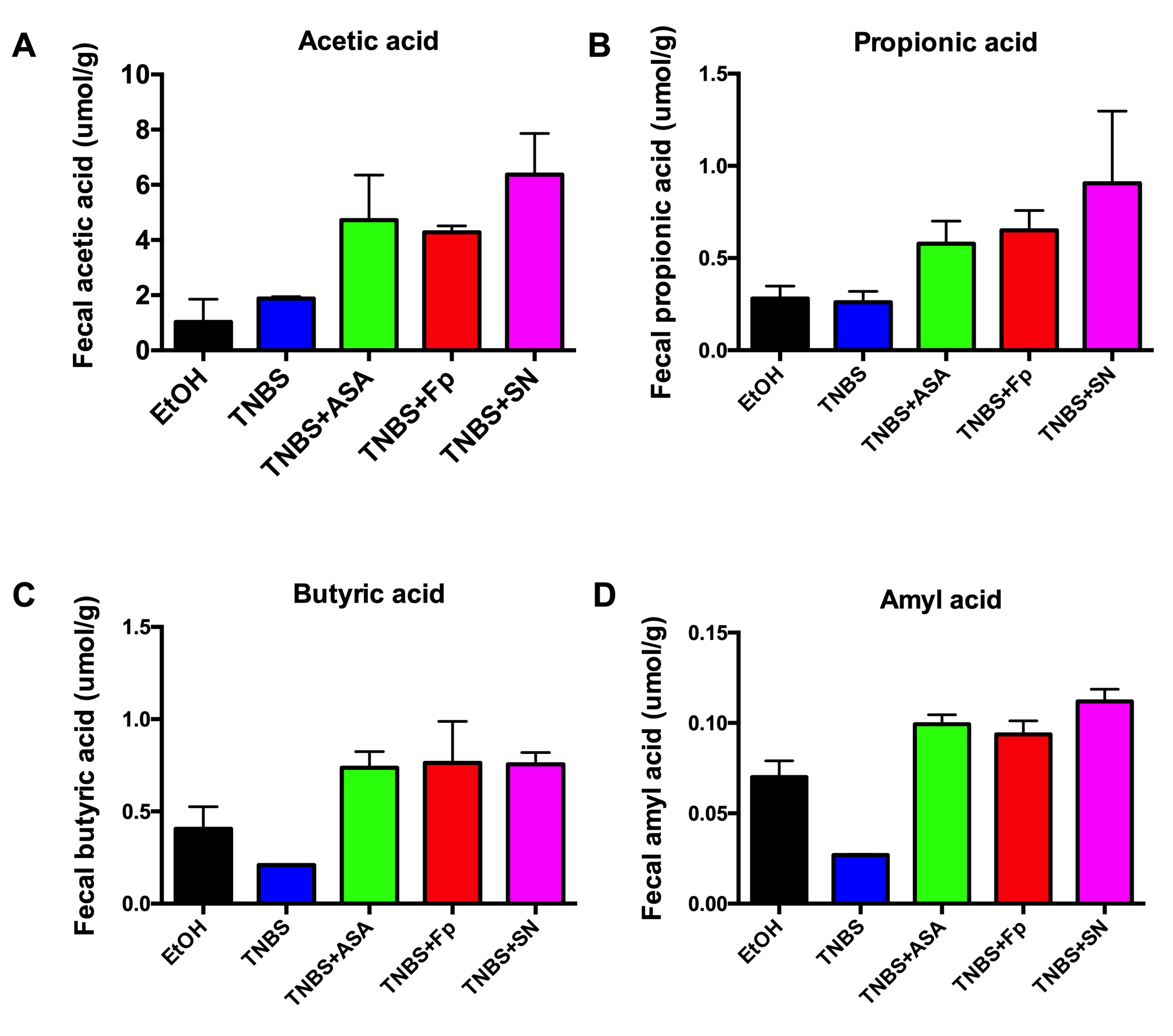

Supplement: Supplementary file 1 — Additional file 1: Figure S1. The fecal SCFAs concentrations including acetic acid (A), propionic acid (B), butyric acid (C) and amyl acid (D). [file 13568_2021_1197_MOESM1_ESM.tiff]
